# Alterations of milk oligosaccharides in mothers with gestational diabetes mellitus impede colonization of beneficial bacteria and development of RORγt^+^ Treg cell-mediated immune tolerance in neonates

**DOI:** 10.1080/19490976.2023.2256749

**Published:** 2023-09-23

**Authors:** Xinke Li, Xixi Ning, Binqi Rui, Yushuang Wang, Zengjie Lei, Da Yu, Feitong Liu, Yanjie Deng, Jieli Yuan, Wenzhe Li, Jingyu Yan, Ming Li

**Affiliations:** a Department of Microecology, College of Basic Medical Science, Dalian Medical University, Dalian, China; b Department of Obstetrics, Dalian Women and Children Medical Center (Group), Dalian, China; c H&H Group, H&H Research, China Research and Innovation Center, Guangzhou, China; d Key Laboratory of Separation Science for Analytical Chemistry, Dalian Institute of Chemical Physics, Chinese Academy of Sciences, Dalian, China

**Keywords:** Gestational diabetes mellitus (GDM), human milk oligosaccharides (HMOs), gut microbiota, RORγt^+^ treg cells, immune tolerance

## Abstract

Gestational diabetes mellitus (GDM) is an increasing public health concern that significantly increases the risk of early childhood allergic diseases. Altered maternal milk glycobiome may strongly affect gut microbiota and enteric-specific Treg cell-mediated development of immune tolerance in GDM infants. In this study, we found that, compared with healthy Chinese mothers, mothers with GDM had significantly lower levels of total and specific human milk oligosaccharides (HMOs) in their colostrum that subsequently increased with extension of lactation. This alteration in HMO profiles significantly delayed colonization of *Lactobacillus* and *Bifidobacterium* spp. in their breast-fed infants, resulting in a distinct gut microbial structure and metabolome. Further experiments in GDM mouse models indicated that decreased contents of milk oligosaccharides, mainly 3ʹ-sialyllactose (3ʹ-SL), in GDM maternal mice reduced colonization of bacteria, such as *L. reuteri* and *L. johnsonii*, in the neonatal gut, which impeded development of RORγt^+^ regulatory T (Treg) cell-mediated immune tolerance. Treatment of GDM neonates with 3ʹ-SL, *Lactobacillus reuteri* (*L. reuteri*) and *L. johnsonii* promoted the proliferation of enteric Treg cells and expression of transcription factor RORγt, which may have contributed to compromising ovalbumin (OVA)-induced allergic responses. *In vitro* experiments showed that 3ʹ-SL, metabolites of *L. johnsonii*, and lysates of *L. reuteri* stimulated differentiation of mouse RORγt^+^ Treg cells through multiple regulatory effects on Toll-like receptor, MAPK, p53, and NOD-like receptor signaling pathways. This study provides new ideas for the development of gut microbiota and immune tolerance in GDM newborns.

## GRAPHICAL ABSTRACT

**Figure:**
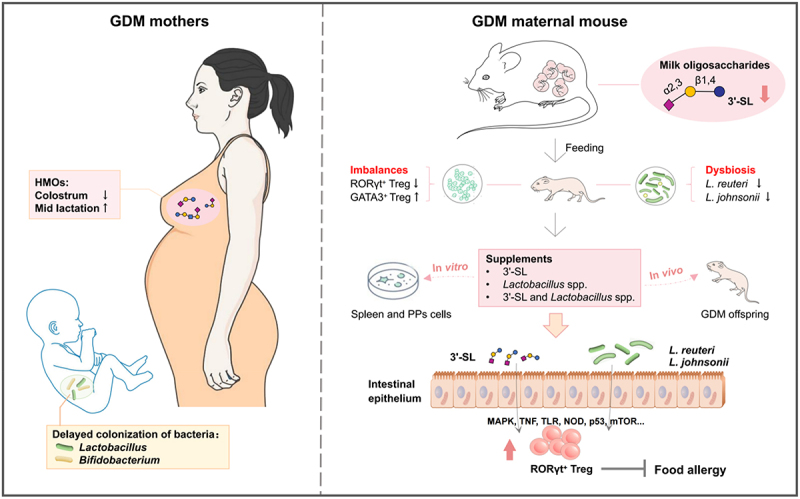

## Introduction

Gestational diabetes mellitus (GDM) is impaired glucose tolerance first diagnosed during pregnancy. It is an increasing public health concern that affects approximately 5–20% of pregnancies. This prevalence has continued to increase during the past few decades and is likely to rise further.^1^ GDM affects both the mother and child with short-term complications, such as preeclampsia, neonatal hypoglycemia, and congenital malformation, whereas long-term complications include maternal type 2 diabetes (T2DM), cardiovascular diseases, obesity, and other metabolic diseases in the offspring.^2^ Notably, GDM is positively associated with early childhood allergic diseases, such as eczema, food allergy, and asthma.^3–5^ Specifically, term infants of pregnancies with GDM have a 7.57-fold increased risk of developing atopic dermatitis, and a 5.91-fold increased risk of allergen sensitization.^3^

Altered microbial communities and the metabolome in GDM neonates affected by vertical transmission of microbiota from their mothers may significantly contribute to dysregulation of immunity, because development of gut microbiota in the early postnatal period parallels maturation of the immune system.^6^ In the second trimester, women with GDM have dysregulated gut microbiota and significantly reduced gut microbial richness compared with healthy pregnant women.^7,8^ The proportions of *Bifidobacterium* spp. and *Lactobacillus* spp. in GDM women are lower than those in women without GDM.^9^ The offspring of GDM mothers show significant differences in gut microbiota composition compared with controls, which is similar to the trend of maternal gut microbiota changes.^7^ In particular, GDM neonates have lower gut microbiota richness,^10,11^ higher relative abundances of proinflammatory bacteria, and significantly lower relative abundances of *Prevotella* spp. and *Lactobacillus* spp.^12^ Intestinal microbiota colonization leads to compartmentalized activation and induction of intestinal regulatory T (Treg) cells, which is critical for successful establishment of intestinal CD4^+^ T cell homeostasis.^13^ Commensal induction of an intestinal Treg cell response is an important intrinsic mechanism to establish immune homeostasis after colonization, which lays the foundation for all subsequent immune responses.^14^ Individual intestinal symbionts promote the proliferation of enteric-specific RORγt^+^ Treg cells by driving the expression of transcription factor RORγt and thus form immune tolerance.^15^ Conversely, intestinal dysbiosis inhibits the expression of RORγt in Treg cells and promotes GATA3^+^ Treg cells with a Th2-like phenotype. These pathological Treg cells cannot inhibit mast cell activation or Th2 cell proliferation, resulting in an impaired intestinal barrier.^16^ Thus, disturbance of gut microbiota in GDM offspring might affect RORγt or GATA3 expression in Treg cells, but no study has tested this hypothesis.

In addition to vertical transmission of microbiota from mothers, the maternal milk glycobiome is another major factor that strongly affects neonatal gut microbiota and immune development.^17^ The most beneficial dietary carbohydrate components in breast milk are human milk oligosaccharides (HMOs) and glycoproteins (HMGs), which are involved in regulating intestinal bacteria colonization and the development of specific and nonspecific immunity of infants.^18–20^ Compared with healthy mothers, mothers with GDM show significant changes in the concentration and glycosylation levels of major glycoproteins such as secretory immunoglobulin A (sIgA) and lactoferrin in transitional milk.^21,22^ Our previous study in mice revealed increases in the levels of fucosylation and sialylation of protein N-glycans in the milk of GDM mice, which might lead to an immune imbalance in offspring by disrupting their gut microbial homeostasis.^23^ However, little is known about the changes of HMOs in GDM human mothers or their effect on neonatal gut microbiota and the development of Treg cell-mediated early-life immune tolerance.

In this study, breast milk samples from healthy and GDM Chinese mothers and fecal samples of their breast-fed infants during various lactation stages were collected to compare maternal HMOs and infantile gut microbiota between the groups, and to evaluate the possible correlation between HMO alteration in GDM mother’s milk on gut microbiota colonization of their breast-fed infants. Subsequently, GDM mouse models were used to further study the effects of alterations in milk oligosaccharides induced by maternal GDM on gut microecology and the development of RORγt^+^ Treg cell-mediated immune tolerance of offspring mice to provide a new direction for prevention of neonatal allergic diseases.

## Results

### Alteration of the HMO profile in GDM Chinese mothers

Between June 2022 and March 2023, 56 healthy infant/mother pairs and 74 GDM infant/mother pairs were selected from the Dalian Women and Children Medical Center (Group), Dalian, China, as research subjects (details in Materials and Methods section). During the study period, maternal age, gestation cycle, the baby’s sex, weight, and gestational age at birth, and the incidence rate of allergic diseases in newborns were recorded (Table 1). Our follow-up survey results showed that the incidence and duration of skin rash, eczema, and diarrhea among GDM infants were markedly higher than those among control infants.

**Table 1.** Mothers and infants information statistics.

		CON (*n* = 57)		GDM (*n* = 74)	*p* Value
**Mother**					
**Lactation period (number of breast milk samples/number of infant fecal samples)** Day 6 Day 42 Day 90		47/31 27/24 5/6		68/37 35/36 11/13	
Fasting blood glucose (mmol/L)		4.54 ± 0.48		5.59 ± 0.73	*p* < .0001***
1 h postprandial blood glucose (mmol/L)		8.99 ± 0.35		10.35 ± 1.52	*p* < .0001***
2 h postprandial blood glucose (mmol/L)		7.77 ± 0.31		9.13 ± 1.62	*p* < .0001***
Age (years)		31.17 ± 3.56		32.04 ± 3.66	*p* = .2077
BMI before pregnancy (kg/m^2^)		22.54 ± 3.49		24.68 ± 4.70	*p* = .0091**
BMI during pregnancy (kg/m^2^)		26.95 ± 2.92		30.09 ± 5.43	*p* = .0004***
Gestational weeks		40.41 ± 1.93		39.97 ± 2.84	*p* = .3573
**Number of pregnancies (n, %)** Primipara Multipara		42 (77.8) 12 (22.2)		61 (84.7) 11 (15.3)	
**Genotype (n, %)** Secretor gene + Secretor gene −		46 (80.7) 11 (19.3)		61 (83.6) 12 (16.4)	
**Infants**					
**Production mode (n, %)** Vaginal Delivery Caesarean Delivery		21 (38.2) 34 (61.8)		29 (40.3) 43 (59.7)	
**Sex (n, %)** Male Female		22 (37.9) 36 (62.1)		24 (58.5) 17 (41.5)	
**Weight (kg)** Birth Day 6 Day 42 Day 90		3.36 ± 0.382 3.62 ± 0.613 4.96 ± 0.698 6.52 ± 0.758		3.53 ± 0.363 3.72 ± 0.407 5.15 ± 0.532 6.98 ± 0.789	*p* = .0790 *p* = .4440 *p* = .2284 *p* = .2689
**Diseases during the intervention period (n, %)** Skin rash (Days) Eczema (Days) Diarrhea (Times)		11 (19.3) 3 (2–3) 5 (4–5) 3 (5–6)		25 (33.8) 8 (2–3) 12 (5–6) 5 (5–6)	*p* = .0071*

*p < .05, **p < .01, ***p < .001.

**Figure 1.**
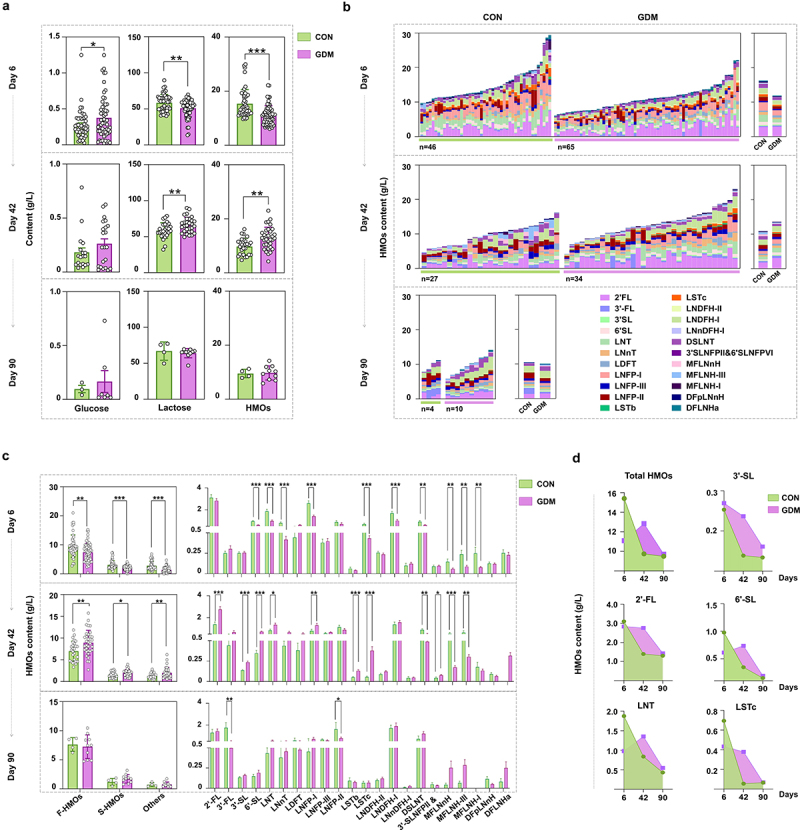
Comparison of HMOs levels between healthy Chinese mothers and mothers with GDM during different lactation stages. (a) the levels of glucose, lactose, and total HMOs in the breast milk of healthy Chinese mothers and mothers with GDM during different lactation stages. (b) comparison of total HMOs in the breast milk of healthy mothers and mothers with GDM at different lactation stages. The left figure shows the level of total and specific HMOs in every sample of each group, and the right figure shows the mean level of total and specific HMOs in each group. (c) the total number of fucosylated HMOs (F-HMOs), sialylated HMOs (S-HMOs) and non-fucosylated neutral HMOs (others) in the breast milk of healthy and GDM mothers was compared in the left figure. On the right is a comparison of individual oligosaccharide levels in breast milk of healthy and GDM mothers. (d) changes of total HMOs and individual oligosaccharides at different time points during lactation. Data were shown as mean ± SEM (*p < .05, **p < .01, ***p < .001, ****p < .0001).

Breast milk samples and infant fecal samples were collected on days 6, 42, and 90 postpartum. The levels of lactose, HMOs, and glucose in breast milk of Chinese mothers were measured by liquid chromatograph combined mass spectrometer (LC-MS). The average level of glucose in milk of GDM Chinese mothers was higher than that of healthy mothers (Figure 1a), especially in colostrum, for which there was a significant difference between the two groups (*p* = .0499), suggesting a continuous effect of GDM. Conversely, the contents of lactose and HMOs in colostrum of GDM Chinese mothers were significantly lower than those of healthy mothers (*p* = .0015, *p* < .0001). However, this pattern was reversed in milk samples collected on day 42. The contents of lactose and HMOs in milk of GDM mothers were significantly increased compared with those of the healthy group (*p* = .0108, *p* = .0018). However, by day 90, lactose and HMO levels showed no significant differences between the groups (All *p* > 0.05). Similarly, comparison of multiple oligosaccharides between the two groups revealed a lower level in the GDM group than in the control group on day 6, a reverse trend on day 42, and comparable levels of multiple oligosaccharides between the two groups on day 90 (Figure 1b). Moreover, the levels of various HMO types varied between the groups during lactation (Figure 1c). The contents of total fucosylated HMOs (F-HMOs), sialylated HMOs (S-HMOs), and non-fucosylated and non-sialylated HMOs (Others) in the colostrum of GDM Chinese mothers were all significantly lower than those of the healthy mothers (*p* = .0013, *p* < .0001, *p* <.0001). The levels of 10 specific HMOs out of the 20 tested major HMO structures, such as 6ʹ-SL, LNT, LNnT, LNFP-I, LSTc, and LNDFH-I, were significantly lower than in the control group. However, by day 42, the levels of various HMO categories were all increased in milk of GDM mothers compared with those in milk of the control mothers. The levels of nine HMOs, including 2ʹ-FL, 3ʹ-SL, 6ʹ-SL, LNT, LNFP-I, LSTb, LSTc, 3ʹ-SLNFP-II and 6ʹ-SLNFP-VI, and DFLNHa, were significantly higher than in the control group, but the levels of other HMOs, including DSLNT, MFLNnH, and MFLNH-III, remained lower than in the controls. By day 90, the levels of various HMO categories were similar between the two groups, but for individual HMOs, the contents of 3ʹ-FL and LNFP-II had declined in the GDM group compared with controls (Figure 1c). In particular, significant changes of total HMOs, 3ʹ-SL, 2ʹ-FL, 6ʹ-SL, LNT, and LSTc in milk between control and GDM groups were observed during lactation (Figure 1d). These results indicated alterations in the HMO profiles of GDM mothers.

Because the mother’s secretory (SE+ or SE-) type, Lewis blood type, and delivery mode significantly affect the HMO composition, we compared HMO levels in breast milk of GDM and healthy Chinese mothers with different Lewis blood types and delivery modes. As shown in Fig. S1, secretory (Se+) Lewis (a-b+) accounted for the largest proportion in all mothers, whereas secretory (Se+) Lewis (a-b-), and non-secretory (Se-) Lewis (a+b-) accounted for small proportions. It is noteworthy that the difference in HMO patterns between Se+ (a-b+) type GDM and healthy mothers was consistent with the general trend shown in Figure 1. Additionally, we found that the changes in HMOs, lactose, and glucose of mothers with different delivery methods were similar to the previous overall trends. Conversely, lower levels of DSLNT, MFLNnH, and MFLNH-III were observed in milk of Cesarean mothers in the GDM group on day 42, suggesting an effect of Cesarean on HMO content (Fig. S2).

### Fecal HMO levels in infants fed by different mothers during lactation

**Figure 2.**
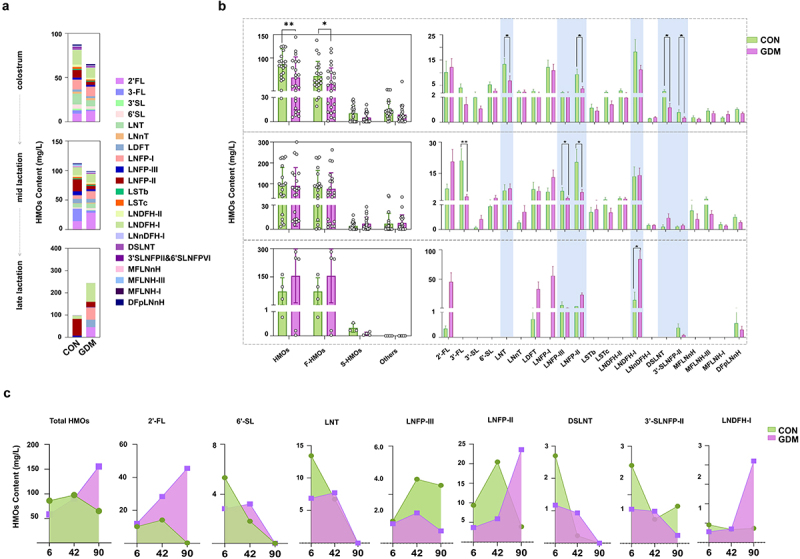
Comparison of fecal HMOs in infants fed by healthy mothers and mothers with GDM during different lactation stages. (a) Comparison of mean fecal oligosaccharides in fecal samples of infants fed by healthy mothers and mothers with GDM at different lactation stages. (b) The left figure compares the total HMOs, fucosylated HMOs (F-HMOs), sialylated HMOs (S-HMOs) and non-fucosylated neutral HMOs (others) in infantle feces. On the right is a comparison of individual HMO levels in infant feces. (c) Changes of total fecal HMOs and individual HMOs at different time points during lactation. Data were shown as mean ± SEM (*p < .05, **p < .01, ***p < .001, ****p < .0001).

To investigate HMO utilization by gut microbiota of infants, we detected the remaining HMOs in fecal samples of infants that were breast-fed by healthy and GDM Chinese mothers (Figure 2a and S3). The content of total and fucosylated HMOs, and specific HMO structures, such as LNT (*p* = .0296), LNFP-II (*p* = .0105), DSLNT (*p* = 0.0416), and 3ʹ-SLNFP-II and 6ʹ-SLNFP-VI (*p* = 0.0433) were significantly lower in day-6 feces of GDM mothers’ infants than those in control infants (Figure 2b). However, by day 42, no significant differences in the fecal contents of total or HMO categories were found between the two infant groups, despite the increased total and individual HMOs in milk of GDM mothers. The fecal contents of 3ʹ-FL (*p* = .0033), LNFP-II (*p* = 0.0218), and LNFP-III (*p* = .0439) in GDM mothers’ infants were even lower than those in the control group, suggesting increased HMO utilization of GDM infants. By day 90, many HMO structures were not found in fecal samples of infants, and the measured HMO contents were comparable between the two infant groups, except for LNDFH-I, which had largely remained in feces of GDM infants compared with that of control infants (*p* = .0390). Unlike the reducing trend of total and specific remaining HMOs in feces of control infants, increasing patterns of total HMOs and 2ʹ-FL were found in feces of GDM infants (Figure 2c). However, other HMO structures, such as 6ʹ-SL, LNT, LSTc, and DSLNT, which were initially lower in feces of GDM infants compared with those of the control group, were increased to similar levels to the control by day 42 and remained higher than those of controls by day 90 (Figure 2c). Additionally, the mother’s secretory type and delivery mode did not affect the general trends of total and specific HMO contents in infant feces described in Figure 2 compared between GDM and healthy control groups (Fig. S4).

### Distinct gut microbial structure of infants fed by GDM mothers and its correlation with maternal HMOs

**Figure 3.**
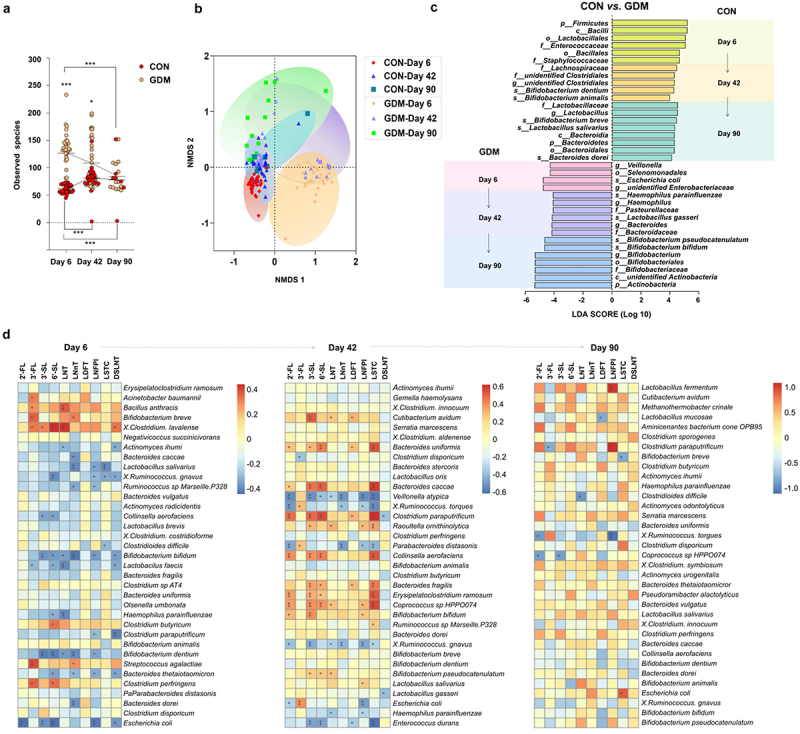
Structural changes of gut microbiota in infants fed by mothers with GDM and its correlation with maternal HMOs. (a) Analysis of α diversity (indicated by the observed bacterial species) of infant gut microbiota. (b) β diversity analysis of infant gut microbiota (indicated by NMDS analysis); (c) Dominant bacteria were significantly different between CON and GDM groups at different time points during lactation (LEfSe analysis); (d) Paired correlation analysis between breast milk oligosaccharides and fecal microbiota abundance of infants. Colour reflects the direction and intensity of Spearman rank correlation coefficient.

We further evaluated the effects of altered HMO patterns of GDM mothers on gut microbiota of their breast-fed infants during lactation. As shown in Figure 3a, the observed total bacterial species in the gut of CON infants was initially as low as 62 ± 8.06 and increased during lactation to 90 ± 12.79 by day 90. Conversely, the total bacterial species observed in the gut of GDM infants on day 6 was 126.4 ± 38.51, which was significantly higher than that of control infants (*p* < .0001), and decreased to 84.31 ± 28.22 by day 90, which was comparable to that in the control group (*p* > .05). β-Diversity analysis by non-metric multidimensional scaling (NMDS) showed that the structure of the gut bacterial community in GDM infants underwent significant fluctuations over lactation stages in contrast to the relatively stable composition in the CON group (Figure 3b). Linear discriminant analysis effect size (LEfSe; Figure 3c) showed that bacterial genera, such as *Lactobacillus* spp. and *Bifidobacterium* spp., had become dominant in gut of control group infants as early as day 6 (*Lactobacillales*) and day 42 (*B. dentium* and *B. animalis*), respectively. Conversely, these bacteria became dominant by day 42 (*L. gasseri*) and day 90 (*B. bifidum* and *B. pseudocatenulatum*) in the gut of GDM infants, suggesting delayed colonization and dominant growth of these bacterial groups. Additionally, the major *Lactobacillus* and *Bifidobacterium* species were distinct between the groups. *L. salivarius*, *B. dentium*, *B. breve*, and *B. animalis* were dominant in control infants, whereas *L. gasseri*, *B. pseudocatenulatum*, and *B. bifidum* were dominant in GDM infants.

We further analyzed the paired correlation between breast milk oligosaccharides and gut microbiota of infants at various lactation stages. Figure 3d shows that the abundance of specific microbial groups in infants was significantly correlated to the levels of various HMOs in their mother’s milk. For example, the abundance of *Bifidobacterium breve* in fecal samples of infants was significantly positively correlated to 3ʹ-FL and LNnT levels in the colostrum of their mothers. By day 42, the abundance of *Bifidobacterium bifidum* in the gut of infants was significantly positively correlated to the levels of 2ʹ-FL, 3ʹ-SL, and LNFPI in their mother’s milk. By day 90, maternal milk LNFPI was positively correlated to the abundance of *Lactobacillus fermentum* in infants. Additionally, we found multiple correlations between various bacterial groups in the gut of infants and various HMOs in the mother’s milk on day 42 compared with days 6 and 90.

**Figure 4.**
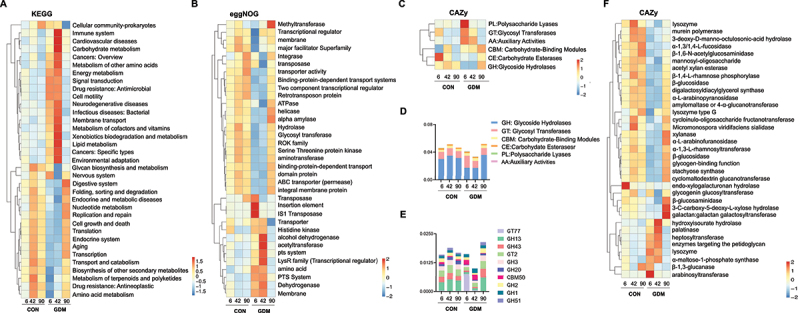
Metagenomic analysis and comparison between fecal microbiota of infants fed by healthy mothers and mothers with GDM. (a) Functional gene expression of fecal bacteria in infants fed by CON and GDM mothers in KEGG database, red for cellular processes, yellow for organismal systems, blue for human diseases, green for metabolism, purple represents environmental information processing and black represents genetic information processing. (b) Expression of functional genes of fecal bacterial populations of infants in the CON and GDM groups in the eggNOG database. (c) Functional gene expression of fecal bacteria in CON and GDM groups in CAZy database. (d) Quantitative analysis of the expression of genes encoding glycoside hydrolases (GHs) in different groups. (e) The expression of major GH and glycosyl transferases (GTs) families in different groups. (f) Heat map analysis of specific genes of CAZy enzymes in different groups.

To further assess the functional differences of fecal microbiota between control and GDM infants, metagenomic sequencing was performed (Figure 4). The resulting genes were analyzed by Kyoto Encyclopedia Genes and Genomes (KEGG), Evolutionary Genealogy of Genes: Non-supervised Orthologous Groups (eggNOG), and Carbohydrate-Active enZYmes (CAZy) databases. In the KEGG database, the control group had more representation of organismal systems, genetic information processing, and metabolism, whereas the microbial gene functions of the GDM group were mainly involved in metabolism, human diseases, and environmental information processing. The similarity between the two groups was high expression on day 42 and weak expression on days 6 and 90 (Figure 4a). In the eggNOG database, the CON group expressed more genes related to nucleic acid modification, such as transposases, helicases, and hydrolases, and genes related to protein transport, such as binding protein-dependent transport systems. The GDM group mainly expressed alcohol dehydrogenase, acetyltransferase, and the phosphotransferase system (Figure 4b). Carbohydrate-active enzymes (CAZymes) act on various substrates. A heat map and quantitative analysis showed that the expression of genes encoding glycoside hydrolases (GHs), which are responsible for hydrolyzing HMOs, was low in the GDM group on day 6 and 42, but was increased by day 90 compared with that in the CON group (Figure 4c,d). We further analyzed the major families of CAZy enzymes and found that the CON group had higher expression levels of genes that belong to GH13, GT2, GH43, GH3, and GH20 families compared with the GDM group on days 6 and 42, but this pattern was reversed by day 90 (Figure 4e). The genes expressed by gut bacteria of CON infants were mainly GHs, such as α-1,3/1,4-L-fucosidase, β-glucosidase, and β-1, 6-N-acetylglucosaminidase, and glycosyltransferases (GTs) such as amylomaltase, or 4-α-glucanotransferase, α-1,3-L-rhamnosyltransferase, and cyclomaltodextrin glucanotransferase. However, the gut bacterial genes expressed in GDM infants were mainly hydrolase heptosyltransferase and hydroxyisourate hydrolase.

### Alteration of oligosaccharide levels in milk of GDM mice during lactation

**Figure 5.**
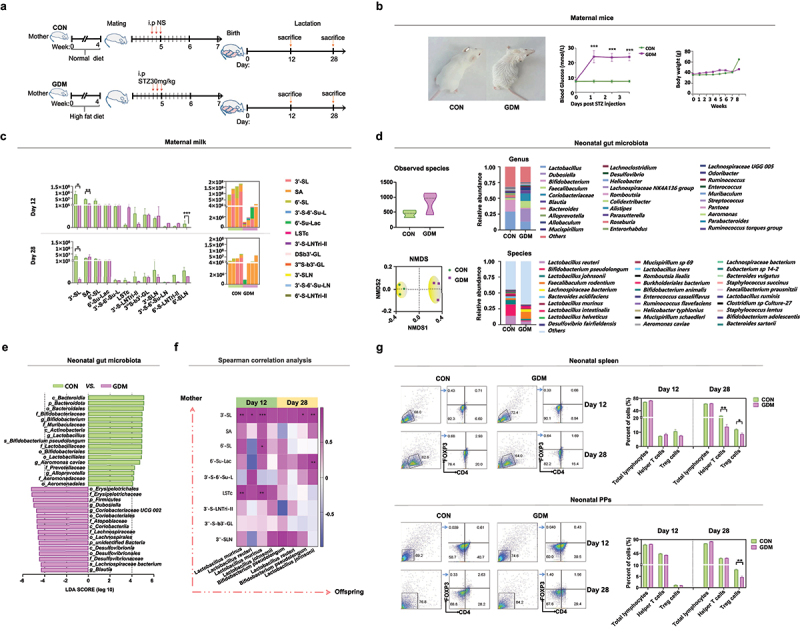
Alterations of milk oligosaccharides in maternal mice with GDM and changes of gut microbiota and Treg cell levels in their offspring. (a) Experimental schema. CON mice (*n* = 10) received normal diets while GDM mice (*n* = 10) were provided high-fat diets before pregnancy. The CON mice were intraperitoneally injected with normal saline while GDM mice were provided with STZ during pregnancy. (b) Photos of the mice fed by normal diets and high-fat diets before pregnancy (left picture). The fasting blood glucose level of the two groups mice post STZ injection (middle picture). Body weights of the two groups mice during lactation (right picture). (c) Histograms of relative intensities of mouse milk oligosaccharide detected by MALDI-TOF MS spectra. From left to right are: total oligosaccharide quantity in different lactation periods, comparison of individual oligosaccharide level, total oligosaccharide level and mean value of each mouse. (d) Gut microbiota offspring fed by CON and GDM mice mothers at different periods during lactation. (e) The bacterial groups that showed significant differences analyzed by the LEfSe. (f) Correlation between gut microbiota in late lactation of offspring and maternal milk oligosaccharides in different lactation periods. Colour and shading reflect direction and strength of Spearman rank correlation coefficients (blue=negative; red=positive; white=no correlation; darker=stronger correlation). (g) The percentage of Treg cells in spleen and PPs of the offspring mice fed by CON and GDM maternal mice in different periods during lactation (each group *n* = 3). Data is shown as mean ± SEM (*p < .05, **p < .01).

To investigate the effect of alterations in milk oligosaccharides of GDM mothers on neonatal gut microbiota, we established a GDM mouse model by a high fat diet (HFD) and streptozotocin (STZ) injection during gestation (Figure 5a). After STZ injection, untidy hair was found in the GDM group (Figure 5b), and the blood glucose level of these mice was significantly higher than that of CON mice. These results suggested successful modeling of GDM. During pregnancy, no significant difference was observed in the body weight of pregnant mice between CON and GDM groups until week 6. However, during week 7, which was 1 week before delivery, the body weight of GDM mice did not increase as much as that in the CON group. We selected two time points, days 12 and 28, which represented the mid and late stages of lactation in mice, to analyze maternal milk and fecal contents of the two groups. Figure 5c and Table 2 show the major oligosaccharides detected in mouse milk by MS methods developed in our previous study,^24^ including 3ʹ-SL, 6ʹ-SL, free SA, and other oligosaccharides. In milk of GDM mice collected on day 12 of lactation, the level of total oligosaccharides was significantly lower than that in CON mice (*p* = .0004), which was mainly caused by reduced levels of 3ʹ-SL (*p* = .0276), SA (*p* = .0014), and 6ʹ-SLN (*p* < .0001). By late lactation (day 28), the level of total oligosaccharides in each group was comparable. However, 3ʹSL content (*p* = .0164) in the milk of GDM mice remained significantly lower than that in CON mice.

**Table 2.** The composition and structures of major mouse milk oligosaccharides detected in this study.

Short name	Composition*	Structure
3’−SL	H2S1	Neu5Aca2-3Galb1-4Glc
6’−SL	H2S1	Neu5Aca2-6Galb1-4Glc
3’-S-6’-Su-L	H2S1Su1	Neu5Aca2-3 Gal(6Su)b1-4Glc
6’-Su-Lac	H2Su1	Gal(6Su)b1-4Glc
LSTc	H3N1S1	Neu5Aca2-6Galb1-4GlcNAcb1-3Galb1-4Glc
3’-S-LNTri-II	H2N1S1	Neu5Aca2-3GlcNAcb1-3Galb1-4Glc
DSb3’−GL	H3S2	Neu5Aca2-3Galb1–3(Neu5Aca2–6)Galb1-4Glc
3’‘S-b3’−GL	H3S1	Neu5Aca2-3Galb1-3Galb1-4Glc
3’−SLN	H1N1S1	Neu5Aca2-3Galb1-4GlcNAc
3’-S-6’-Su-LN	H1N1S1Su1	Neu5Aca2-3 Gal(6Su)b1-4GlcNAc
6’-S-LNTri-II	H2N1S1	Neu5Aca2–6(GlcNAcb1–3)Galb1-4Glc

*N, HexNAc; S, Neu5NAc; Su, SO3H.

### Colonization of gut microbiota in GDM neonatal mice correlates to altered maternal milk oligosaccharides and gut microbiota

Because of the alterations in milk oligosaccharides of GDM mice, we further investigated whether these changes affected early colonization of microbes and immune development in their offspring. 16S rRNA gene sequencing indicated that the observed gut bacterial species in GDM neonates were slightly increased compared with those in CON neonates, but without a significant difference (*p* > .05). However, the gut microbial structure of GDM neonates indicated by NMDS analysis was significantly distinct from that of CON neonates (Figure 5e). The relative abundances of *Lactobacillus* spp., such as *L. reuteri* and *L. johnsonii*, were also reduced in the gut of GDM neonates. LEfSe shown in Figure 5f indicates that gut microbiota of neonatal mice in the CON group was characterized by abundant *Lactobacillus*, *Bifidobacterium*, and *Alloprevotella*, whereas in the gut of GDM neonates, bacterial genera such as *Dubosiella*, *Coriobacteriaceae* UCG002, and *Blautia* were dominant. Spearman correlation between gut microbiota of offspring and the abundance of milk oligosaccharides of maternal mice during lactation was analyzed, and the abundance of *L. murine*, *L. reuteri*, and *B. pseudolongum* in the gut of offspring was positively correlated to the levels of 3ʹ-SL, 6ʹ-SL, and LSTc in the milk of mothers in mid lactation. Furthermore, in late lactation, the abundance of these bacteria was highly positively correlated to the maternal 3ʹ-SL level.

### Development of treg cells in GDM offspring

Early colonization of gut microbiota affects development of the host immune system, especially intestinal mucosal immune cells. As a T helper cell subset, Treg cells play an important role in controlling the immune balance.^13^ To determine whether the alteration in gut microbiota of GDM offspring affected their immune development and responses, we analyzed the proportion of Treg cells in the spleen and Peyer’s patches (PPs) of offspring fed by control or GDM maternal mice at mid and late lactation by flow cytometry. As shown in Figure 5g, although the total lymphocyte counts of the groups were comparable, the proportion of splenic T helper (CD4^+^ T) cells was significantly lower in offspring fed by GDM mothers in late lactation (day 28) than in GDM neonates (*p* = .0058). The proportion of Treg (CD4^+^FoxP3^+^) cells in the spleen of GDM-fed neonates was significantly lower than that of the CON group in late lactation (*p* = .0341). The percentages of total lymphocytes (*p* = .0001) and Treg cells (*p* = .0039) in PPs of neonatal mice were significantly lower in GDM offspring during late lactation compared with that of CON offspring. These results suggested that the alteration of neonatal gut microbiota had affected the development of Treg cells in GDM offspring.

### GDM neonates are susceptible to ovalbumin (OVA)-induced allergic responses

**Figure 6.**
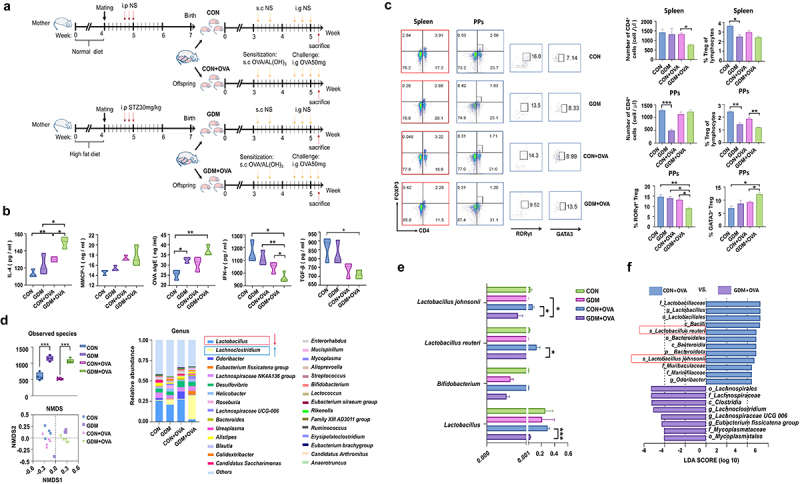
Alteration of immune responses and the abundance of gut *Lactobacillus* spp. In GDM offspring with OVA-induced FA. (a) Schematic of FA protocols. The offspring fed by CON/GDM mouse was injected with 100 μg ovalbumin (OVA) and 2 mg Aluminum hydroxide adjuvant (AL(OH)3). The challenge was performed after 7 days: the neonatal mice were gavaged 50 mg OVA for 4 times. There were four groups in total: CON group (n = 4), CON+OVA group (n = 4), GDM group (n = 5) and GDM+OVA group (n = 5). (b) Cytokines in the serum of offspring after OVA sensitization. (c) The flow cytometry analysis of the proportion of lymphocytes in the offspring after OVA sensitization. Red represents spleen, and blue represents PPs. (d) Gut microbiota of offspring after OVA sensitization. (e) Compared the relative abundance of Bifidobacterium and Lactobacillus in the four groups. (f) The bacterial groups that showed significant differences analysed by the LEfSe. Data are shown as mean values α SEM (*p < .05, **p < .01).

To investigate the effect of the altered intestinal microbiota and immune status in GDM offspring on development of immune tolerance, we established a food allergy (FA) model in offspring by OVA/AL(OH)_3_ sensitization and OVA challenge (Figure 6a). The levels of circulating cytokines and immune parameters in serum of offspring were measured by enzyme-linked immunosorbent assays (ELISAs) before and after OVA challenge. The serum concentrations of IL-4, MMCP-1, and OVA sIgE were higher in FA offspring compared with the control group (Figure 6b). Notably, post-OVA challenge, GDM offspring had a significantly higher serum concentration of IL-4 than offspring fed by CON mice (*p* = .0115). Conversely, GDM offspring had a significantly lower concentration of INF-γ (*p* = .0478) than CON offspring, suggesting disrupted development of immune tolerance in GDM offspring. Considering the important roles of Treg cells in mediating immune tolerance, we further examined the frequencies of Treg cells and related subset cells of offspring with OVA-induced FA (Figure 6c). Flow cytometry showed that the proportions of total Treg cells in the spleen of GDM, CON+OVA, and GDM+OVA groups showed a downregulated trend compared with the CON group. Moreover, the proportion of Treg cells in PPs was significantly lower in offspring fed by GDM mice (*p* = .0029). Post-OVA challenge, the proportion of Treg cells in GDM-fed offspring was even lower compared with that in CON offspring (*p* = .0078). As an important subset of Treg cells,^25^ the proportion of RORγt^+^ Treg cells in the GDM+OVA group was significantly lower than that in the CON+OVA group (*p* = .0235), whereas the proportion of GATA3^+^ Treg cells in the GDM+OVA group was significantly higher than that in the CON+OVA group (*p* = .0141).

### Gut lactobacillus spp. decrease significantly in GDM offspring with OVA-induced FA

We further compared neonatal gut microbiota before and after OVA challenge. Figure 6d shows that OVA challenge increased bacterial species and altered the microbial structures of CON and GDM offspring. The abundance of *Lachnoclostridium* was significantly increased in offspring fed by GDM maternal mice post-OVA challenge, while *Lactobacillus* spp. and *Bifidobacterium* spp. were obviously reduced in this group (all *p* < .0001, Figure 6d, right), although the latter accounted for only a small proportion of neonatal gut microbiota. At the species level, the relative abundances of *L. reuteri* and *L. johnsonii* were significantly lower in the GDM+OVA group than in the CON+OVA group (*p* = .0195, *p* = .0389, Figure 6e). LEfSe analysis confirmed that *L. reuteri* and *L. johnsonii* were significantly less abundant in the GDM+OVA group than in the CON+OVA group (Figure 6f), suggesting an important role of *Lactobacillus* spp. in regulating immune tolerance of neonatal mice.

### *Supplementation of*L. johnsonii, L. reuteri *and 3ʹ-SL in GDM offspring improves the proportion of intestinal Treg cells*

**Figure 7.**
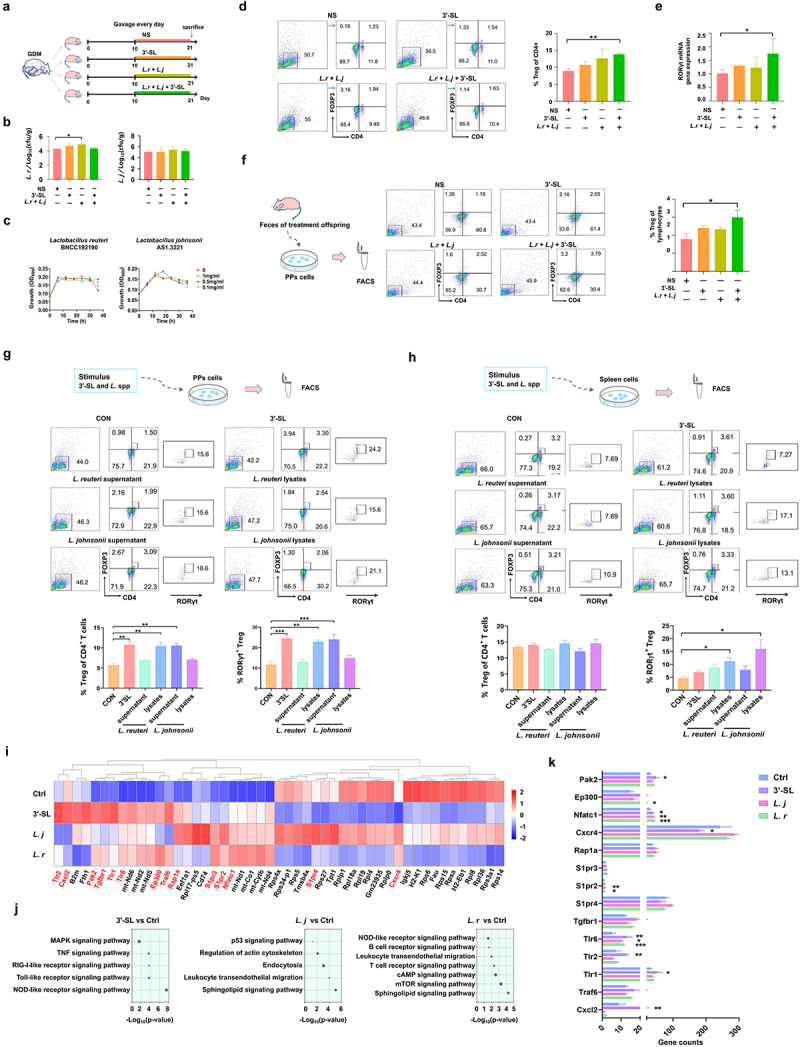
*L. johnsonii*, *L. reuteri* and 3’-SL improve the development of RORγt^+^ Treg cells in mice. (a) Scheme of GDM offspring treated with 3’-SL, *L. johnsonii* and *L. reuteri* (*Lactobacillus* spp.) or 3’-SL in the combination with the two *Lactobacillus* strains. (b) The content of *Lactobacillus* spp. In feces of GDM offspring in different treatment groups detected by qPCR. (c) The growth curves of *L. johnsonii* and *L. reuteri* when cultured in liquid media supplemented with 3’-SL of different concentrations as the sole carbon source. (d) Comparison of splenic Treg cell populations in GDM offspring received different treatment. (e) Comparison of the RORγt gene expression of the gut in the GDM offspring received different treatment. (f) Flow cytometry analysis of Treg cells populations after vitro stimulation of PPs lymphocytes by fecal metabolites of GDM offspring with different treatments. (g and h) Flow cytometry analysis of the proportion of RORγt^+^ cells after vitro stimulation of PPs and splenic lymphocytes by supernatant or cell lysates of *L. reuteri* and *L. johnsonii*, or 3’-SL. (g) PPs. (h) spleen. (i) Heatmap of differential gene clustering for all groups. (j) KEGG pathway enrichment analysis. The size of the dot represents the number of genes annotated to the KEGG pathway. (k) The major up-regulated genes were in the KEGG pathway. NOD-like receptor signaling pathway (Cxcl2, Traf6), Toll-like receptor signaling pathway (Tlr1, Tlr2, Tlr6), MAPK signaling pathway (Tgfb1), Sphingolipid signaling pathway (S1pr4, S1pr2, S1pr3), leukocyte transendothelial migration (Rap1a, Cxcr4), cAMP signaling pathway (Nfatc1, Ep300), T cell receptor signaling pathway (Pak2). Data is shown as mean ± SEM (*p < .05, **p < .01, ***p < .00).

To further investigate the effect of alterations in milk oligosaccharides of GDM maternal mice on the immune tolerance of offspring, we gavaged GDM neonatal mice with *L. reuteri* and *L. johnsonii* or 3ʹ-SL continuously for 10 days during lactation (Figure 7a) and detected changes in gut bacteria and Treg cell proportions of neonatal mice. The contents of *L. reuteri* and *L. johnsonii* in feces were quantified by qPCR with species-specific primers post-intervention (Figure 7b, Table 3). Compared with neonates in the NS group, the contents of *L. reuteri* and *L. johnsonii* were increased in intervention groups, especially the *L*. spp. intervention group (*p* = .0142). However, intervention by combined 3ʹ-SL and *Lactobacillus* spp. administration did not increase these bacteria in the neonatal gut, suggesting that 3ʹ-SL was not the direct carbon source for *L. reuteri* and *L. johnsonii*. This result was confirmed by *in vitro* cultivation of *L. reuteri* and *L. johnsonii in* 3ʹ-SL-supplemented medium (Figure 7c).

**Table 3.** Characteristics of quantitative real-time PCR primers.

Gene name	Sequence	Product (bp)	Ref
RORγt-F	5′-CAAGTCATCTGGGATCCACTAC-3′	94	65
RORγt-R	5′-GCAGGAGTAGGCCACATTACA-3′		
β-Actin-F	5′-AGCCATGTACGTAGCCATCC-3′	222	66
β-Actin-R	5′-GCTGTGGTGGTGAAGCTGTA-3′		
*Lactobacillus reuteri*-F	5′-CCCTTTCTTTCAGATCCACAGG-3′	245	63
*Lactobacillus reuteri*-R	5′-TGGTTGTAGAGAAGGGGACG-3′		
*Lactobacillus johnsonii*-F	5′-CACTAGACGCATGTCTAGAG-3′	127	64
*Lactobacillus johnsonii*-R	5′-AGTCTCTCAACTCGGCTATG-3′		

The splenic Treg cells of neonates post-intervention were also assessed by flow cytometry. As shown in Figure 7d, although the total lymphocyte counts among groups were, the percentage of Treg (CD4^+^FoxP3^+^) cells in the spleen of neonates supplemented with combined *L. reuteri*, *L. johnsonii*, and 3ʹ-SL was significantly higher than that in control neonates (*p* = 0.0012). Additionally, we found a significant promoting effect of combined 3ʹ-SL and *Lactobacillus spp*. (*L. spp*.) intervention on RORγt mRNA expression in the intestinal tissue of neonates (Figure 7e, *p* = 0.0205). These data suggest that supplementation of this combination promoted the development of intestinal RORγt^+^ cells in neonatal mice, which is essential for the development of intestinal mucosal immune tolerance.^26^

To further analyze the interaction between the changes in the gut microbiome and the immune response of GDM neonates, we extracted fecal metabolites from neonatal mice supplemented with *Lactobacillus* spp. or 3ʹ-S, and applied them to T lymphocytes obtained from mouse PPs *in vitro* (Figure 7f). Flow cytometry showed that the percentage of Treg cells was increased by fecal metabolites from GDM mice supplemented with *L. spp*. or 3’-SL compared with the normal saline group. Furthermore, the percentage of Treg cells in the combined intervention group was significantly higher than that in the control group (*p* = 0.0464).

### *Metabolites of* L. reuteri *and* L. johnsonii *promote differentiation of mouse RORγt^+^ Treg cells in vitro*

To determine whether *L. reuteri, L. johnsonii*, and 3ʹ-SL had direct effects on RORγt^+^ Treg cell development, we isolated lymphocytes from the PPs and spleens of mice and incubated them with 3ʹ-SL and the supernatant or cell lysate of *L. reuteri* and *L. johnsonii in vitro* (Figure 7g). After incubation, the number of Treg cells, especially RORγt^+^ Treg cells, was much higher following stimulation with *L. reuteri* lysates, *L. johnsonii* supernatant, and 3ʹ-SL (*p* = .0029, *p* = .0080), whereas *L. reuteri* supernatant and *L. johnsonii* lysates had no promoting effects. Stimulation of RORγt^+^ Treg cell differentiation by *Lactobacillus* spp. lysate was found more pronounced in splenic lymphocytes (Figure 6h, *p* = .0112, *p* = .0279).

To clarify the mechanisms of 3ʹ-SL and *Lactobacillus* spp. in promoting the development of RORγt^+^ Treg cells, we performed RNA-seq analysis of stimulated splenic cells to investigate gene-level regulation by *Lactobacillus* spp. metabolites and 3ʹ-SL in mouse lymphocytes. Compared with the untreated control group, 3ʹ-SL treatment increased expression of Toll-like receptor (TLR), C-X-C motif chemokine 2 (Cxcl2), Ep300, TNF receptor associated factor 6 (Traf6), and transforming growth factor beta 1 (Tgfb1), and treatment with *L. reuteri* and *L. johnsonii* increased expression of sphingosine 1-phosphate receptor (S1pr), Ras-associated protein 1A (Rap1a), C-X-C chemokine receptor type 4 (Cxcr4), nuclear factor of activated T cell cytoplasmic 1 (Nfatc1), and p21-activated kinase 2 (Pak2) (Figure 7i). KEGG analysis showed that the main pathways promoted by 3ʹ-SL intervention were Toll-like receptor, Nucleotide oligomerization domain (NOD)-like receptors (NLRs), and mitogen-activated protein kinase (MAPK) signaling pathways, whereas stimulation by *L. reuteri* and *L. johnsonii* primarily upregulated the Sphingolipid signaling pathway, Leukocyte transendothelial migration, cAMP signaling pathway, and T cell receptor signaling pathways (Figure 7j). Furthermore, we observed a significant increase in expression of genes, such as Cxcl2 (NOD-like receptor signaling pathway), Toll-like receptor (Toll-like receptor signaling pathway),^27^ Tgfb1 (MAPK signaling pathway),^28^ S1pr receptor (Sphingolipid signaling pathway),^29^ Nfatc1 (cAMP signaling pathway),^30^ and Pak2 (T cell signaling receptor pathway),^31^ compared with the control group (Figure 7k). These genes were all involved in regulating and supporting of Treg cell development promoted by 3ʹ-SL and *L*. spp.

## Discussion

Human milk glycans provide a broad range of carbon sources for the gut microbiota of infants.^32,33^ HMOs promote a healthy microbial composition and enhance intestinal mucosal maturation and the epithelial barrier function to modulate immune functions in offspring.^34,35^ GDM is associated with a high prevalence of allergic diseases in offspring.^3–5^ It alters the glycosylation pattern of N-glycans in human milk, and thus affects the gut microbiota composition of offspring.^36,37^ However, little is known about the effects of altered HMOs of GDM in mothers on the gut microbiota and development of immune tolerance in their offspring. In the present study, we systematically characterized changes in HMO patterns in Chinese GDM mothers during various stages of lactation and evaluated the possible effects on gut microbiota and immune responses in their infants.

Our human cohort study revealed that the contents of total and most HMOs in colostrum of Chinese GDM mothers were significantly lower than those in healthy controls, increased gradually to a comparable or even higher level than those in controls by day 42, and then remained at a similar level to those in controls on day 90. Saben et al.^38^ investigated the association between third-trimester glucose homeostasis in healthy women and the HMO composition at 2 months postpartum. Third trimester fasting plasma glucose and insulin were associated with HMOs such as 3ʹ-SL and DSLNT in a secretor-dependent manner. Wang et al.^39^ reported that mothers with GDM had lower levels of S-HMOs, especially 3ʹ-S, compared with healthy secretor mothers. These studies supported alterations in the HMOs pattern of GDM mother’s milk. However, because of different collection times of milk samples and the various detection methods between our study and others, repetition and larger samples are needed to confirm these findings and elucidate clinical consequences. Nonetheless, we did find a delayed pattern of HMO content in colostrum of GDM mothers. Why GDM mothers had less total and specific HMOs in their colostrum and a greater HMO amount later at day 42 remains poorly understood. One possibility is that obese women are more likely to experience delayed onset of lactogenesis. In our study cohort, the BMIs of mothers in the GDM group before and during pregnancy were significantly higher than those of the control group (Table 1). Even after adjusting for confounding variables, a consistent association has been found between maternal obesity and delayed lactogenesis.^40^ Disturbances in glucose metabolism may underlie the association between maternal obesity/GDM and delayed onset of lactogenesis.^41^ Neubauer et al. revealed a significantly lower mean lactose concentration at day 2 postpartum among insulin-dependent diabetic mothers compared with non-diabetic mothers.^42^ This is consistent with our finding that the lactose content was significantly lower in GDM colostrum compared with the healthy group (Figure 1). Insulin is an important factor in regulating lactation, and has essential roles in secretory differentiation, secretory activation, and mature milk production.^43^ Notably, insulin plays a crucial role in the transcription of milk protein genes,^44,45^ including ɑ-lactalbumin, which is a main component of the lactose synthase complex and controls the rate of lactose synthesis in mammary glands.^46^ Therefore, the insulin resistance of GDM mothers may have hindered lactose biosynthesis from glucose by disturbing ɑ-lactalbumin production. As a result, HMO production was also delayed because the HMO synthesis in lactating mammary glands is catalyzed by specific glycosyltransferases using lactose as the acceptor.^47^ However, whether GDM affects the expression of glycosyltransferase genes remains unclear, which may be a good direction for future research.

In contrast to the different levels of HMOs between GDM and healthy controls, no significant difference was found in the remaining content of oligosaccharides in infant feces between the two groups at various stages of lactation. This suggested that the HMO-utilizing ability of gut microbiota in infants fed by GDM mothers was weak in the early stage of lactation, despite the higher bacterial diversity in this group of infants than that of CON group infants, which may be affected by vertical transmission from their mothers. Changes have been found in the gut microbiota of offspring reared by GDM mothers, which may be affected by altered microbiota in GDM mothers.^7–12,48^ However, the colonization and deductive patterns of various microbial communities, especially *Lactobacillus* and *Bifidobacterium* spp., which are pivotal for the development of infant health, together with various lactation periods remain unclear. Our results clearly showed a delay in the colonization of these bacteria in the gut of infants fed by GDM mothers. As shown in Figure 3, in infants fed by CON mothers, *Lactobacillus* and *Bifidobacterium* spp. had colonized and became dominant as early as days 6 and 42 of lactation, respectively. Conversely, in the gut of infants fed by GDM mothers, *Lactobacillus* and *Bifidobacterium* spp. became dominant by days 42 and 90, respectively. These results suggested that, in addition to vertical transmission of altered gut microbiota in GDM mothers, the low levels of breast milk oligosaccharides in GDM mothers during early lactation may have strongly disrupted the proliferation of *Lactobacillus* and *Bifidobacterium* spp. in the infantile gut. In support of this, as the HMO levels of GDM mothers increased by day 42, the proliferation of these bacteria and their ability to utilize HMOs also increased significantly. Additionally, the abundances of other bacterial groups in the infant gut were also significantly correlated to HMO contents in the mother’s milk, suggesting a strong effect of HMOs on shaping neonatal gut microbiota and functions. Significant differences in the function of infant fecal microbiota were detected between GDM and controls. The bacterial functions in fecal samples of the control group mainly involved genetic information processing and metabolism, whereas in the GDM group, the functions of fecal bacteria were mainly involved in human disease and environmental information processing. In summary, our cohort study showed that changes in HMO patterns in GDM mothers had significantly delayed the colonization and metabolism of gut microbiota in infants. However, because of the low number of breast milk and infant fecal samples collected at the 90-day time point, our results at this time point are limited. Therefore, it is necessary to conduct large-scale studies to evaluate the long-term effects of GDM breast milk HMOs on the gut microbiota of newborns.

Children of GDM mothers have a high incidence of allergic diseases, which is closely related to disruption of gut microbiota and immune development during infancy. To study the effect of GDM breast milk oligosaccharides on the development of gut microbiota and immune tolerance in offspring, we established a GDM model in mice. Although mouse models complement human studies to a certain extent, the types and content of oligosaccharides in mouse milk are less abundant based on our previous studies,^23,24^ and only contain acidic sialylated oligosaccharides and other structures. However, we found that the contents of total oligosaccharides and 3ʹ-SL in the milk of GDM mice were significantly lower than those in CON mice at the middle and late stages of lactation, which reflects the possible effect of GDM on milk oligosaccharide synthesis similar to our human study. Breast milk oligosaccharides play important roles in promoting early-life colonization of bacteria in the neonatal gut, which contributes significantly to the maturation of their immune system.^49^ Therefore, we further analyzed the main differences in gut microbiota and the proportions of various lymphocytes between CON and GDM offspring. Among the gut microbiota of neonates, *Bifidobacterium* and *Lactobacillus* were more abundant in CON offspring than in GDM offspring. This was confirmed by Spearman correlation, in which *Bifidobacterium* and *Lactobacillus* of offspring gut microbiota were significantly correlated to breast milk oligosaccharides, especially 3ʹ-SL. Although we only assessed a late lactation timepoint, which is a limitation of this study, our results support that 3ʹ-SL is an important factor affecting the composition of the offspring’s gut microbiota, especially dominant growth of *Bifidobacterium* and *Lactobacillus* spp. Flow cytometry showed that the proportions of Treg cells in the spleen and Peyer’s patches of GDM offspring were significantly lower than those of CON offspring during late lactation. Treg cells are responsible for maintaining immune tolerance and controlling excessive immune responses. Therefore, this result implies that the immune functions of GDM offspring would be affected.

Gut microbiota plays a vital role in the development and regulation of the immune system. Microbiota disturbance is associated with the development of multiple allergic diseases in infants.^50–53^ Therefore, we established a food allergy model to further explore the effect of changes in the gut microbial composition on development of immune tolerance in mice. Our results showed that the levels of proinflammatory factors,^54^ such as IL-4, IFN-γ, and TGF-β, were significantly higher in the GDM+OVA group than in the CON+OVA group. Additionally, the proportion of Treg cells, especially RORγt^+^ Treg cells, in PPs was significantly decreased in the GDM+OVA group. Beneficial gut microbiota suppress neonatal food allergy by inducing expression of the transcription factor RORγt in Treg cells in a MyD88-dependent manner.^55^ Conversely, increased GATA3 expression and IL-4 secretion lead to dysregulated FA responses, dietary allergen-specific IgE responses, and impaired barrier integrity.^56^ This suggests that offspring with GDM are at higher risk of developing food allergies. Next, we analyzed changes in gut microbiota of the food allergy model mice. The abundance of *Lactobacillus* in the GDM+OVA group was significantly lower than that in the other groups, and the difference between *L. reuteri* and *L. johnsonii* was more significant. Therefore, we believe that *L. reuteri* and *L. johnsonii* have a significant effect on the development of immune tolerance in offspring. To explore the regulatory effects of *L. reuteri* and *L. johnsonii* on Treg cell-mediated development of immune tolerance, we conducted *in vitro* and *in vivo* experiments. GDM offspring were treated with 3ʹ-SL and *Lactobacillus* spp., which significantly increased the proportion of Treg cells and upregulated RORγt expression. RORγt is involved in maintenance of mucosal tolerance in the gut,^15^ and RORγt^+^ Treg cells are directly involved in the protective mechanism against food allergy.^57^ This suggests that administration of milk oligosaccharides and *Lactobacillus* spp. to GDM offspring promotes the establishment of immune tolerance in newborn offspring. Administration of probiotics to murine FA models enhances immune tolerance by promoting Treg cell expansion.^58,59^ Our in vitro experiments showed that the fecal bacterial metabolites of mice treated with 3ʹ-SL, *L. reuteri*, and *L. johnsonii* significantly increased the proportion of Treg cells among PP-derived lymphocytes. However, it was unclear whether *Lactobacillus* spp., which was enriched in the control group of neonatal mice, but depleted in GDM neonates, directly affected their immune responses. Therefore, we isolated PPs and the spleen from mice and incubated them with *L. reuteri* and *L. johnsonii* supernatants and lysates. The results showed that the proportions of Treg and RORγt^+^ Treg cells in PPs were significantly increased after stimulation with *L. reuteri* lysate, *L. johnsonii* supernatant, and 3ʹ-SL, whereas the spleen was more responsive to *Lactobacillus* spp. lysate. RNA-seq of stimulated spleen cells provided further insights into the gene-level regulation of *Lactobacillus* spp. metabolites and 3ʹ-SL in lymphocytes. *Lactobacillus* spp. lysates and metabolites as well as 3ʹ-SL contributed to Treg development to varying degrees. 3ʹ-SL mainly upregulated some genes of Toll-like receptor, NOD-like receptor, and MAPK signaling pathways. The Toll-like receptor 1-2-6 axis and Cxcl2 conduct a Treg amplification signal and induce Treg cell proliferation.^27^ Treg cell-derived TGF-β1 regulates allergy and autoimmunity.^28^ *Lactobacillus* spp. metabolites upregulated the sphingolipid signaling pathway, leukocyte transendothelial migration, and cAMP signaling pathway. S1pr is involved in immune regulation, and Nfatc1 and Pak2 support the phenotype of Foxp3^+^ Treg cells and maintain the function of suppressive Treg cells.^30,31^ The maintenance of immune tolerance by Treg cells is critical to prevent allergic and autoimmune diseases.^60^ Thus, our results suggested that supplementation of 3’-SL, *L. reuteri*, and *L. johnsonii* synergistically promoted the proliferation of intestinal RORγt^+^ Treg cells through multiple pathways, which in turn contributed to establishment of immune tolerance to OVA stimulation in offspring. However, further study is needed to analyze the specific metabolites of *L. reuteri* and *L. johnsonii*, after utilizing 3ʹ-SL through metabolomics research, so as to reveal the regulatory role of Treg cell differentiation through activating different signaling pathways.

In conclusion, this study revealed alterations in milk oligosaccharide profiles of GDM mothers and evaluated the possible effect of such an alteration on the development of neonatal gut microbiota and RORγt^+^ Treg cell-mediated immune tolerance. Our findings may provide a rationale for studies in human populations, particularly aimed at early supplementation by microecological preparations that combine HMOs and *Bifidobacterium*/*Lactobacillus* spp. in children to maintain microecological equilibrium and prevent allergic diseases.

## Material and methods

### Subjects and sample collection

A subset of 56 healthy infant/mother pairs and 74 GDM mother/infant pairs was selected from the Dalian Women and Children Medical Center (Group), Dalian, China, as research subjects, and their informed consent was obtained. Participants were recorded at about 34 weeks of pregnancy, excluding pregnant women with high blood pressure, diabetes, metabolic disease and who had used antibiotics and microecological agents in the last month. During the study period, maternal age, gestation cycle and the baby’s sex, weight and gestational age at birth were recorded (Table 1). Breast milk samples and fecal samples were collected at day 6, 42, and 90 postpartum. For the collection of breast milk samples, subjects had milk from one breast fully pumped into a bottle, then transferred the milk from the bottle to a sterile polypropylene tube for immediate storage at −20°C, transported the sample to the laboratory on dry ice within 1 week, and stored at −80°C for future use. Fecal samples are collected on the same day and stored in the same way as breast milk samples. The diagnostic criteria of GDM is that any of the three blood glucose levels measured during pregnancy (fasting/1 h postprandial/2 h postprandial blood glucose) >10.0 mmol/L can be diagnosed as GDM. Infant fecal samples were collected on the morning of each of the days when milk samples were collected. To eliminate the effect of the formula feeding on gut microbiome of infants, only those who received breast-feeding were enrolled. Infants who received antibiotics, probiotics, medicines or solid foods because of diseases or lack of breast milk were excluded during the study period. Parents were instructed to immediately store the samples in −20°C until the time when the samples were transported on dry ice to the laboratory, where the samples were stored at −80°C before processing. The study was approved by the ethics committees of Dalian Women and Children Medical Center (Group), Dalian, China (No. 2022018).

### Detection of oligosaccharides in milk and feaces

The breast milk was taken out of the −80°C freezer and placed on ice until thawed and mixed completely. 1 mL of breast milk sample was centrifuged at 8,000 rpm at 4°C for 15 min to remove the upper layer of lipids, and 2 mL of ethanol was added to remove the protein at 8,000 rpm at 4°C for 10 min. The obtained supernatant was diluted 10 times for the detection of HMOs. For fecal HMOs detection, 100 mg of fecal samples were added to 1 mL sterile water, vortexed into a homogenate, and placed in a 4°C shaker overnight. The supernatant was centrifuged at 8,000 rpm for 15 min in a centrifuge at 4°C. 2 mL ethanol was added to the supernatant and centrifuged at 8,000 rpm for 10 min at 4°C. The resulting supernatant was diluted 10 times for the detection of HMOs. HMOs in breast milk and infant feaces were analyzed using the LC-MS system, in which the Agilent Series 1290 LC unit was used in conjunction with the Agilent Series 6540 time-of-flight mass spectrometer. Dry gas temperature 350°C, flow rate 8.0 L/min. Agilent Mass Hunter qualitative analysis software was used to identify and quantify HMOs. The mobile phase used to separate the standard mixture consists of water and acetonitrile. The solvent gradient was carried out at a flow rate of 0.2 mL/min: 0–40 min, 20–50% water. The loading volume of the sample was 2 µL.^61^ For the detection of mouse milk oligosaccharides, a high-sensitivity online solid-phase extraction and HILIC coupled with electrospray tandem mass spectrometry method that was previously developed by our group was adopted.^24^

### Extraction and sequence analysis of fecal bacteria 16S rRNA gene

The microbial genome DNA from fecal samples of the infants and mice was extracted using the E.Z.N.A. stool DNA kit (Omega Bio-tek, Inc.) according to the manufacturer’s instructions. A Nanodrop 2000 spectrophotometer was used to evaluate the purity (A260/A280) and concentration (ng/μL) of isolated DNA, the qualified samples are stored in aliquots and placed in the −80°C freezer for later use. After the quality of DNA isolations were examined, they were used to amplify the gut bacterial V3-V4 region. The PCR primers used were as follow: 341 F: 5′-CCTAYGGGRBGCASCAG-3′; 806 R: 5′-GGACTACNNGGGTATCTAAT-3′. Sequences of the 16S rDNA amplicons were sequenced on an Illumina NovaSeq PE250 and 250 bp paired-end reads were generated (Novogene Bioinformatics Technology Co. Ltd, Beijing). The sequences obtained after quality control analysis were used in the present analysis. A total of 9,179,972 high-quality sequences were obtained, with each sample assembled into 65,106 effective tags with a maximum of 69,961 and a minimum of 60,296, which were clustered into 768 operational taxonomy units (OTUs) were uploaded to QIIME (Quantitative Insights Into Microbial Ecology, v1.8.0) for further study. The OTUs of representative sequences at a similarity cut off of 97% and their relative abundance (alpha-diversity) were used to calculate Shannon and other indexes by UCLUST. The abundance and diversity of the OTUs (beta-diversity) were examined using Principal coordinates analysis (PCoA) with unweighted UniFrac analysis in R software. The statistical significance of the separation among groups was assessed by the linear discriminant analysis effect size (LEfSe) method based on linear discriminant analysis scores exploited, which used the nonparametric factorial Kruskal – Wallis and Wilcoxon rank sum test to identify key OTUs for separating different treatment groups at a significance level of 0.05. Correlations between the gut microbiota of offspring and the maternal milk oligosaccharides were tested using Spearman rank correlation and visualized using a heatmap. The correlation coefficient (r) indicates the degree of correlation between two variables. The higher the absolute value of the *r* value means the higher the correlation. *P* value < .05 was considered as a significant correlation.

### Metagenomic analysis of infant fecal microbiota

All the samples were paired-end sequenced on the Illumina platform (insert size 350 bp, read length 150 bp) at the Novogene Bioinformatics Technology Co., Ltd. After quality control, the reads aligned to the human genome alignment with SOAP2 (Version 2.21, parameters: -s 135, -l 30, -v 7,-m 200,-x 400) were removed. There was a total of 241,525 prediction genes after the original redundancy removal, of which 211,558 (87.59%) genes were able to match the KEGG database. There were 193,128 (79.96%) genes that could match eggNOG database and 9,555 (3.96%) genes that could match CAZy database. Unigenes was aligned to each functional database (blastp, evalue ≤ 1e-5) by DIAMOND software; alignment results filtered: for the alignment results for each sequence, the identity values were chosen to be larger than the minimum identity values required by the database to ensure the reliability of the results of the annotation of the resistance gene; then, the relative abundance and number of genes at different functional levels were calculated from the comparison results; finally, starting from the abundances table of each classification level, the number of annotated genes, relative abundances and abundance cluster heatmap were displayed.

### Animals study

Twenty female and 20 male Institute of Cancer Research (ICR) mice aged 8 weeks were obtained from the specific-pathogen-free laboratory of Dalian Medical University, China. The female mice were randomly divided into two groups (*n* = 10 in each group): the healthy control group (CON group) and gestational diabetes group (GDM group). Before the experiment, venous blood of mouse tail was collected to determine the fasting blood glucose (FBG), with 3‐5 mmol/L as the normal blood glucose value. the CON group were given standard diet, and the GDM group were fed with high-fat diet (20% protein, 20% carbohydrate, 60% fat) for 4 weeks.^23,62^ Starting from the fifth week, the vaginal smear method was used to determine the estrus cycle of mice. The female and male mice in the early stage of estrus were mated. The vaginal opening and litter were checked on the next morning to observe whether there was vaginal suppository in mice. The observed vaginal suppository in mice was regarded as the first day of pregnancy. The GDM group of mice were intraperitoneally injected with streptozotocin (STZ, each: 30 mg/kg) solution in saline for continuous 3 days, and mice in the CON group were injected with the same dose of saline. Three days after the last injection, the random blood glucose was detected for 3 consecutive days, the level >16.7 mmol/L was considered as successful establishment of GDM mouse model. The body weight of the female mice during pregnancy was measured weekly using an electrical balance. The female mice were fed in separate cages after parturition, half of the maternal mouse and their offspring were executed on the 12th day (mid-lactation) and 28th day (late-lactation), respectively. The mammary gland of mice was collected to analyze the changes of oligosaccharides in breast milk of GDM mothers at different stages, the immune levels of mice in spleen and PPs were analyzed by flow cytometry, the feces were collected to compare the differences of gut microbiota. The methods of maternal oligosaccharide detection and gut microbiota of offspring detection were consistent with those of HMOs and infant fecal 16S rRNA gene high-throughput sequencing analysis. All of the animal experiments were performed using protocols approved by the committee for animal care and use at Dalian Medical University (SYXK [Liao] 2018–0002), and were conducted according to the Guide for the Care and Use of Laboratory Animals (NIH publication no. 8023).

### Flow cytometric analysis

The spleen and PPs of the neonatal mice were ground up in phosphate-buffered saline (PBS). Then the cells were obtained through a 200-mesh filter and centrifuged at 2,500 rpm for 5 min. The supernatants were discarded and the lysis buffer was added to remove red blood cells and wash them with PBS buffer twice. 1 × 10^6^ cells were isolated and used for flow cytometry with a fluorescence-activated cell sorter (FACS). Cells were incubated with anti-mouse CD16/CD32 monoclonal antibody (MAb) to block Fcγ receptors and then stained on ice for 30 min with combinations of MAbs. Next, the cells were incubated with anti-mouse CD4 monoclonal antibody (MAb) for 30 min on ice for staining. Furthermore, the cells were incubated with the rupture fluid in the dark for 1 h. This step was for the monoclonal antibody (MAb) to enter the cell and bind to intracellular factors. Finally, the cells incubated with anti-mouse Foxp3 and anti-mouse RoR gamma(t) and Gata-3 monoclonal antibody (MAb) for half an hour on ice and protected from light. The MAbs used in this study were anti-mouse CD16/CD32, fluorescein isothiocyanate (FITC)-labeled anti-mouse CD4, allophycocyanin (APC)-labeled anti-mouse Foxp3, PE-labeled anti-mouse RoR gamma(t) and Gata-3, all purchased from BD Biosciences. The flow cytometry assay was performed on a FACS-Calibur (Becton Dickinson, Mountain View, CA) and analyzed using FlowJo software (Tree Star).

### Induction of food allergy (FA) in offspring mice

After 4 weeks of lactation, the offspring were separated into cages and feed with the normal diet. mice were sensitized on the first day of the fifth week, and the offspring mice fed by mothers in the CON and GDM group were further divided into four groups: the CON group, CON+OVA group, GDM group and GDM+OVA group. To sensitize, each mouse was immunized by subcutaneous injection with 100 µg ovalbumin (OVA) in 100 µL saline mixed with 2 mg aluminum hydroxide adjuvant. The first subcutaneous injection was followed by a second injection 2 days apart. the CON and GDM group was administered i.p. saline injections of the same amount. The challenge was performed after 7 days: the mice were gavaged 50 mg OVA (50 mg OVA dissolved in 200 µL of normal saline) once every other day for a total of 4 times. Mice in the control group were given the same amount of saline as control. Mice were starved for 3–4 h before each intragastric challenge to ensure that the OVA antigen could quickly pass through the stomach without being destroyed by gastric acid. Mice were sacrificed 1 hour after the last challenge and the degree of allergy was assessed.

### Detection of serum cytokines

Blood samples were collected and centrifuged at 3,000 rpm for 15 min to obtain serum. The levels of serum OVA specific immunoglobulin E (OVA sIgE), interleukin 4 (IL-4), mast cell protease (MMCP-1), Interferon-γ (IFN-γ) and transforming growth factor-β (TGF-β) were measured using enzyme-linked immunosorbent assay (ELISA) (Jiangsu Meibiao Biotechnology Co., Ltd, China). The absorbance was measured at 450 nm using a computer-interfaced microplate reader (BioRad, Houston, TX).

### Extraction and sequence analysis of 16S rRNA gene from mice feces

The microbial genome DNA from fecal samples of the offspring mices was extracted using the E.Z.N.A. stool DNA kit (Omega Bio-tek, Inc.) according to the manufacturer’s instructions. After DNA isolation quality detection, amplification was carried out. The fecal DNA of different times collected by all the offspring mice samples revealed 2,014,051 high-quality filtered reads, which assembled into 64,534 effective tags per samples. The maximum is 68,168 which the minimum is 61,411. These clean tags were clustered into 565 OTUs with 97% identity. According to the results of OTUs clustering, species annotations were made on the representative sequence of each OTU, and the community composition of each sample was counted at each classification level. The richness and diversity of microbial community in the group were evaluated by Alpha diversity. Non-metric multidimensional scaling (NMDS) was used to analyze Beta diversity (inter-group difference analysis).

### Culture of bacterial strains and growth detection

The standard strain *Lactobacillus reuteri* BNCC192190 (from BeNa Culture Collection) and *Lactobacillus johnsonii* AS1.3221 (from MingZhouBio Culture Collection) were used in this study. They were routinely grown at 37°C in liquid MRS medium. Growth was monitored by measuring the optical density (OD) at 600 nm. Three technical replicates were performed for each strain.

### *Intervention of* L. reuteri, L. johnsonii *and 3’-SL to the offspring mice*

The offspring of GDM maternal mice were divided into four groups and gavaged with saline, *L. reuteri* and *L. johnsonii* (10^7^ cfu in 15 µL saline per mouse) or 3’-SL (0.1 mg in 15 µL saline per mouse), each mouse in the combined intervention group received *L. reuteri* (10^7^ cfu in 5 µL saline), *L. johnsonii* (10^7^ cfu in 5 µL saline) and 3’-SL (0.1 mg in 5 µL saline). Supplementation was started on the 10th day after birth, once a day until weaning on the 21st day.

### RNA extraction and quantitative real-time polymerase chain reaction (qPCR)

The colon of mice was ground into a tissue homogenate. Total RNA was extracted and purified by TansZol (TransGen Biotech, Beijing, China) according to the manufacturer’s instructions. Quality and purity of the extracted RNA was assessed by a Nanodrop 2000 spectrophotometer. The prepared RNAs from all samples were reverse-transcribed using a cDNA synthesis kit (TransGen Biotech, Beijing, China). Standard qPCR was carried out in CFX96 Touch™Real-Time PCR Detection System (BioRad, Irvine, CA, USA) using the *PerfectStart*^R^ Green qPCR SuperMix (BioRad, Irvine, CA, USA). The sequences of primers are shown in Table 3. The β-actin was used as housekeeping gene.

The primers used to quantify *L. reuteri* and *L. johnsonii* were those described by Fernández *et al*. ^63^ and Furet *et al*.^64^ The final volume was 10 µl, containing 1× PerfectStart® Green qPCR SuperMix (Applied TransGen Biotech, Beijing, China), 0.3 µM of each primer and 1 µl of each DNA sample. qPCR conditions were 95°C for 5 min, 39 amplification cycles and melting curve analysis from 60°C to 95°C. The cycle consisted of a first denaturation step at 95°C for 20 s, annealing for 30 s (58°C for *L. reuteri* and 60°C for *L. johnsonii*) and extension for 30 s at 72°C. For quantitative analysis, the Cq of each sample was compared to the Cq of a standard curve constructed with dilutions of the strain’s genomic DNA. Taking into account the size of the genomes of these species, bacterial chromosome copy numbers in the standard curve were calculated. The genome sizes used for the calculations were 2.0 Mb for *L. reuteri* and 1.99 Mb for *L. johnsonii*.

### Culture and stimulation of mouse lymphocytes in vitro

To analyze the effect of gut microbiome changes on Treg cell proliferation in the PPs of mice, we extracted fecal metabolites from GDM mice fed with *Lactobacillus* and milk oligosaccharide, and co-cultured with T lymphocytes obtained from PPs *in vitro*. Briefly, 0.25 g of mouse feces and 1 ml of PBS were mixed and centrifuged at 13,000 rpm for 30 min. The obtained supernatant was filtered through a 0.45 µm filter and a 0.22 µm filter in sequence. The filtered liquid, considered fecal metabolites, was diluted 10-fold and 20 µL was added to 1 ml of RPMI1640 medium containing 1 × 10^6^ cells. All cells were cultured in a cell incubator at 37°C, 5% CO_2_ for 6 hours.

To detect the effects of *L. reuteri, L. johnsonii* and 3’-SL on the proliferation of Tregs in the PPs of mice, we isolated total lymphocytes from the PPs of the neonatal mice and co-cultured them with 3’-SL, *L. reuteri* and *L. johnsonii* supernatant and cell lysates, respectively. Briefly, 1 mL of the *L. reuteri* and *L. johnsonii* culture broth (OD_600_ = 1.5) was centrifuged at 3,000 rpm for 15 min. The supernatant was regarded as bacterial secretion. The pellet was resuspended in 1 mL of PBS for ultrasonic crushing to release the cellular components. After that, the mixture was centrifuged at 3,000 rpm for 15 minutes, and the supernatant after filtering with a 0.22 µm filter was used as a lysate of *Lactobacillus*. The supernatant and lysate of *L. reuteri* and *L. johnsonii* were diluted ten-fold and 20 µL was added to 1 ml RPMI1640 medium containing 1 × 10^6^ cells. An equal amount of 3’-SL in the milk oligosaccharide group was added to a final concentration of 1 mg/ml. All cells were cultured in a cell incubator at 37°C, 5% CO_2_ for 6 hours.

### RNA-seq experiments and analysis

To test the effects of 3’-SL, *L. reuteri* and *L. johnsonii* on global gene transcription of mouse lymphocytes by RNA-seq. Total lymphocytes were isolated from the spleen of neonatal mice and co-cultured with 3’-SL, *L. reuteri* and *L. johnsonii* supernatant and cell lysate, respectively. Total RNA was extracted and checked for integrity using an Agilent Bioanalyzer 2100 system (Agilent Technologies, CA, USA). Sequencing libraries were generated using the NEBNext® UltraTM RNA Library Prep Kit for Illumina® (NEB, USA) according to the manufacturer’s recommendations. The HiSeq X-TEN/NovaseqS4 PE Cluster Kit (Illumina) was used to cluster different groups of samples on the cBot Cluster Generation System according to the manufacturer’s instructions. After cluster generation, library preparations were sequenced on an Illumina NovaSeq 6000 platform and 150bp double-terminal readings were generated. Differentially expressed mRNAs were selected according to Log2 (fold change) >1 or p-value <0.05 by DESeq2 R package (1.20.0). Kyoto Encyclopedia Genes and Genomes (KEGG) analysis was performed for respectfully expressed gene.

### Statistical analysis

All data were expressed as mean ± SEM (*n* ≥ 3). Statistical analysis of the experimental data was performed with the help of GraphPad Prism 8.1 (Graph Pad Software, La Jolla, CA, USA) to compare differences. A non-parametric *t* test was performed between the two groups. One-way analysis of variance and Tukey’s test were used between multiple groups to evaluate whether there were statistical differences between the groups. Spearman rank correlation analysis was employed to assess correlation between the gut bacteria of offspring and the milk oligosaccharides of maternal mice. *p* values of <0.05 are considered statistically significant (*p < .05; **p < .01; ***p < .001).
